# Exploring complementary and competitive relations between non-communicable disease services and other health extension programme services in Ethiopia: a multilevel analysis

**DOI:** 10.1136/bmjgh-2022-009025

**Published:** 2022-06-23

**Authors:** Azeb Gebresilassie Tesema, David Peiris, Rohina Joshi, Seye Abimbola, Fasil Walelign Fentaye, Alula M Teklu, Yohannes Kinfu

**Affiliations:** 1 The George Institute for Global Health, Faculty of Medicine, University of New South Wales, Sydney, New South Wales, Australia; 2 School of Public Health, College of Health Sciences, Mekelle University, Mekelle, Ethiopia; 3 School of Population Health, Faculty of Medicine, University of New South Wales, Sydney, New South Wales, Australia; 4 The George Institute for Global Health, New Delhi, India; 5 School of Public Health, Faculty of Medicine, University of Sydney, Sydney, New South Wales, Australia; 6 Monitoring, Evaluation, Research, and Quality Improvement (MERQ), Ethiopia office, MERQ Consultancy PLC, Addis Ababa, Ethiopia; 7 Department of Public Health, Faculty of Health, University of Canberra, Canberra, ACT, Australia; 8 Department of Health Science Metrics, University of Washington, Seattle, Washington, USA

**Keywords:** Health policies and all other topics, Health services research, Health systems, Community-based survey, Health systems evaluation

## Abstract

**Background:**

Ethiopia has recently revitalised its health extension programme (HEP) to address the rising burden of non-communicable diseases (NCDs). We examined the effects of existing essential HEP services on the uptake of NCD preventive services.

**Methods:**

We applied a mixed-effect non-linear model with a logit link function to identify factors associated with a community resident’s probability of receiving NCD prevention services through the HEP. The data were drawn from the Ethiopian HEP assessment Survey conducted in all regions. The analysis included 9680 community residents, 261 health extension workers (HEWs), 153 health posts, 119 health centres, 55 districts and 9 regions, which we combined hierarchically into a single database.

**Results:**

In the 12 months before the survey, 22% of the sample population reported receiving NCD preventive service at least once. The probability of receiving NCD prevention service increased by up to 25% (OR=1.25, CI 1.01 to 1.53) if health centres routinely gathered NCD data from health posts and by up to 48% (OR=.48, CI 1.24 to 1.78) if they provided general (ie, non-NCD specific) training to HEWs. NCD preventive service uptake also increased if the HEW held level IV qualification (OR=1.32, CI 1.06 to 1.65) and lived in the community (OR=1.24, CI 1.03 to 1.49). Conversely, if facilities delayed general performance reviews of HEWs by a month, uptake of NCD prevention services decreased by 6% (OR=0.94, CI 0.91 to 0.97). We observed that better HIV/AIDS programme performance was associated with a lower uptake of NCD preventive services (OR=0.15, CI 0.03 to 0.85).

**Conclusion:**

Despite efforts to improve NCD services through the HEP, the coverage remains limited. A strong HEP is good for the uptake of NCD preventive services. However, integration requires a careful balance, so that the success already recorded for some existing programmes is not lost.

**WHAT IS ALREADY KNOWN ON THIS TOPIC**

- Ethiopia’s primary healthcare focused health extension programme (HEP) has been a guiding framework for its health sector development for the past two decades, with non-communicable disease (NCD) preventive service being added in 2016.
- Previous studies have only examined the impacts of the HEP such as maternal and child health, HIV/AIDS and tuberculosis.

**WHAT THIS STUDY ADDS**

- Despite ongoing efforts to improve NCD preventive services through the HEP, coverage of NCD services is still limited, more so for underserved communities.
- NCD services, is influenced by improved health information system, health worker training and timely performance appraisal practices.
- HIV/AIDS service delivery appears to compete with NCD preventive service provision.

**HOW THIS STUDY MIGHT AFFECT RESEARCH, PRACTICE OR POLICY**

- A strong HEP is good for the uptake of NCD preventive services, but integration with existing programmes may not be straightforward. It requires a carefully balanced approach, so that the success already recorded for some existing programmes is not lost.
- Furthermore, strengthening overall training, information management system and building trust between the community and the HEWs are all essential for a successful HEP, just as they are essential for individual services within the HEP and for integrating NCD preventive services.
- A range of complementary effects point to the need for an overall strong HEP capable of delivering existing services as a prerequisite for successful integration of new services.

## Background

Non-communicable diseases (NCDs) account for one-third of all deaths in Ethiopia.^1–3^ Rapid urbanisation, epidemiological transition, population ageing and lifestyle changes are anticipated to compound the burden of and unmet need for NCD services in the country even further. This poses a significant threat to Ethiopia’s healthcare system, which, thus far, has been focused on tackling HIV, malaria, tuberculosis (TB) and maternal and child health (MCH). The mismatch has been more significant for communities living far from health facilities.^4^

Rising community expectations for better and inclusive services and the growing NCD burden in Ethiopia led to a comprehensive National NCD Prevention and Control Strategic Action Plan. The plan of action focused on behavioural risk factors for NCDs, with a clear strategy to address them through the country’s health extension programme (HEP).^5^ Implemented in 2003, the HEP is a community-based strategy geared towards delivering essential health promotion, disease prevention and selected curative health services at the community and health postlevel. The programme was revitalised in 2016/2017, introducing mental health and NCDs as additional packages, primarily focusing on cardiovascular disease, diabetes, cancer and chronic respiratory diseases.^6 7^

Through the optimised HEP, the scope of work for Health Extension Workers (HEWs) has been expanded over and above their pre-existing role and included NCD prevention and promotion activities. HEWs—10th-grade school graduates who have received a 1-year training in primary healthcare (PHC) before being deployed as salaried civil servants in health posts in their respective villages—were initially responsible for implementing 16 preventive, promotive and selected curative packages focused on four programmatic areas: family health, disease prevention and control, hygiene and environmental sanitation and health education and communication.^6 8 9^

Under the optimised HEP, HEWs role for NCDs included: awareness creation of NCD risk factors, promoting of NCD prevention activities through health education, undertaking community screening for early detection of NCDs, referring patients with more complex health needs to health centres and collecting and interpreting NCD data in the community.^7 10–12^ A preservice training programme had been launched to support the HEWs new mandate and the newly introduced packages. This created career transition opportunities and allowed several HEWs to transition from level III to level IV qualifications while also gaining NCD content as part of the curriculum.^7^

Although research on community health workers’ (CHWs) role in NCD prevention is still evolving, the evidence to date suggests that CHWs can be effective in NCD prevention in low-income and middle-income countries.^13–18^ Several studies have demonstrated an association between CHW programme performance and health system factors, such as training and availability of NCD guidelines.^13–18^ Furthermore, evidence suggests that community-level attributes, such as sociodemographic characteristics and perception towards health workers and programmes on offer, contribute to the success of CHW-based NCD programmes.^17 19^

However, despite growing evidence on CHW programmes’ role in strengthening health systems and improving key health outcomes, including NCDs,^14–18^ existing studies in Ethiopia and elsewhere have several limitations. First, none of the studies from Ethiopia focused on NCD programme performance delivered at the HEP level. Second, although new packages are often added over existing services, no other research has empirically investigated the complementarity and competition between NCD service provision and other services. Similarly, not enough is known, if any, health system inputs—such as supervision and performance appraisal—HEW attributes and community perceptions towards the HEP or HEWs have the same effect on NCDs as in other programmes.^19 20^ This study uses nationally representative data to investigate the utilisation of NCD preventive services through the HEP in Ethiopia. Our specific aims are to examine (1) the effect of health system inputs and processes, (2) the association between HEWs’ and community residents’ characteristics and NCD uptake and (3) the complementarity and competition between NCD and other essential preventive services.

## Methods

### Study setting

Ethiopia’s HEP, primarily implemented by HEWs, is part of the country’s PHC strategy to improve health service coverage and health outcomes. The PHC operates under a three-tier system: primary, secondary and tertiary care. The primary care component includes primary hospitals, health centres and health posts (the lowest service delivery point at the village level). The PHC unit comprises five satellite health posts and a referral health centre. In each health post, an average of two HEWs serve around 3000–5000 people in their catchment area. In 2019, there were 3790 health centres,^21^ 17 587 health posts and 39 878 HEWs across the country. HEWs are posted within a health post and receive technical support and supervision from the nearby health centre.^4 7^ A designated HEW manages health posts, and all HEW working within the health posts are responsible for delivering all essential health service packages, including NCD services.

At the time of data collection, Ethiopia is divided into nine regions and two city administrations (ie, Addis Ababa and Dire Dawa). Each region, in turn, is subdivided into zones, and zones are divided further into districts, locally known as woredas.^22^ Woredas are the primary administrative unit in Ethiopia’s decentralised system and have a council and separate operational and sectoral offices, including one for health, known as a woreda health office.^22^ A group of kebeles—each of which has around 5000 inhabitants—constitute a single woreda. Kebele is the country’s lowest administrative unit and provides administrative support to the health post. A detailed description of Ethiopia’s health system has been reported elsewhere.^23^

### Study design

Data from the Ethiopian HEP assessment Survey (EHAS), conducted from 1 October 2018 to 31 October 2019, were used for this study.^4^ The EHAS was commissioned by the Ministry of Health (Ethiopia) and carried out by the Monitoring, Evaluation, Research and Quality Improvement consultancy group. The national assessment covered all regions and agro-ecological zones in the country and employed qualitative and quantitative methods. Only the quantitative component was used for this study.

The quantitative survey was carried out following a hierarchical multistage sampling design to identify the study Woredas, HPS, HCs and community members in all the nine regions in the country, excluding the two city administrations. In the first stage, the Woredas in each region were classified into pastoralist and agrarian communities based on their primary means of livelihood. Subsequently, six kebeles were chosen randomly from each woreda, and the facility assessment surveys were conducted on all health centres and health posts located in the selected kebeles. The household survey was conducted only on three kebeles randomly chosen out of the original six kebeles. The total sample included 62 woredas, 179 rural health centres, 343 health posts, 584 HEWs and 12 868 respondents living in 7122 rural households.^4^ The woreda level data were supplied by the woreda health office manager or their representative, while health centre directors provided the data on health centres. The head of the health post, a HEW, was responsible for providing the data related to the health post.

Additionally, each HEW in the selected area provided data on their age, gender, qualification, year of service, other HEW attributes and perceptions or views on the HEP and existing support system. Finally, service utilisation patterns, including NCDs, were obtained from male and female community respondents from the selected areas. The survey also recorded participant characteristics and views towards the HEP and the HEWs working in their communities.

In all, this study captured 9680 community residents nested with 261 HEWs, 153 health posts, 119 health centres and 55 woredas. The analytic sample included only those observations with complete data at all levels of the data collection system. Additional material on the underlying survey is available elsewhere.^4^

### Conceptual framework

Our analysis focuses on the utilisation of HEWs-based NCD prevention services. The selection of covariates affecting service uptake was guided by data availability and conceptual and empirical evidence on the role of CHWs in the provision of various community preventive services in resource-limited settings.^24^

**Figure 1:**
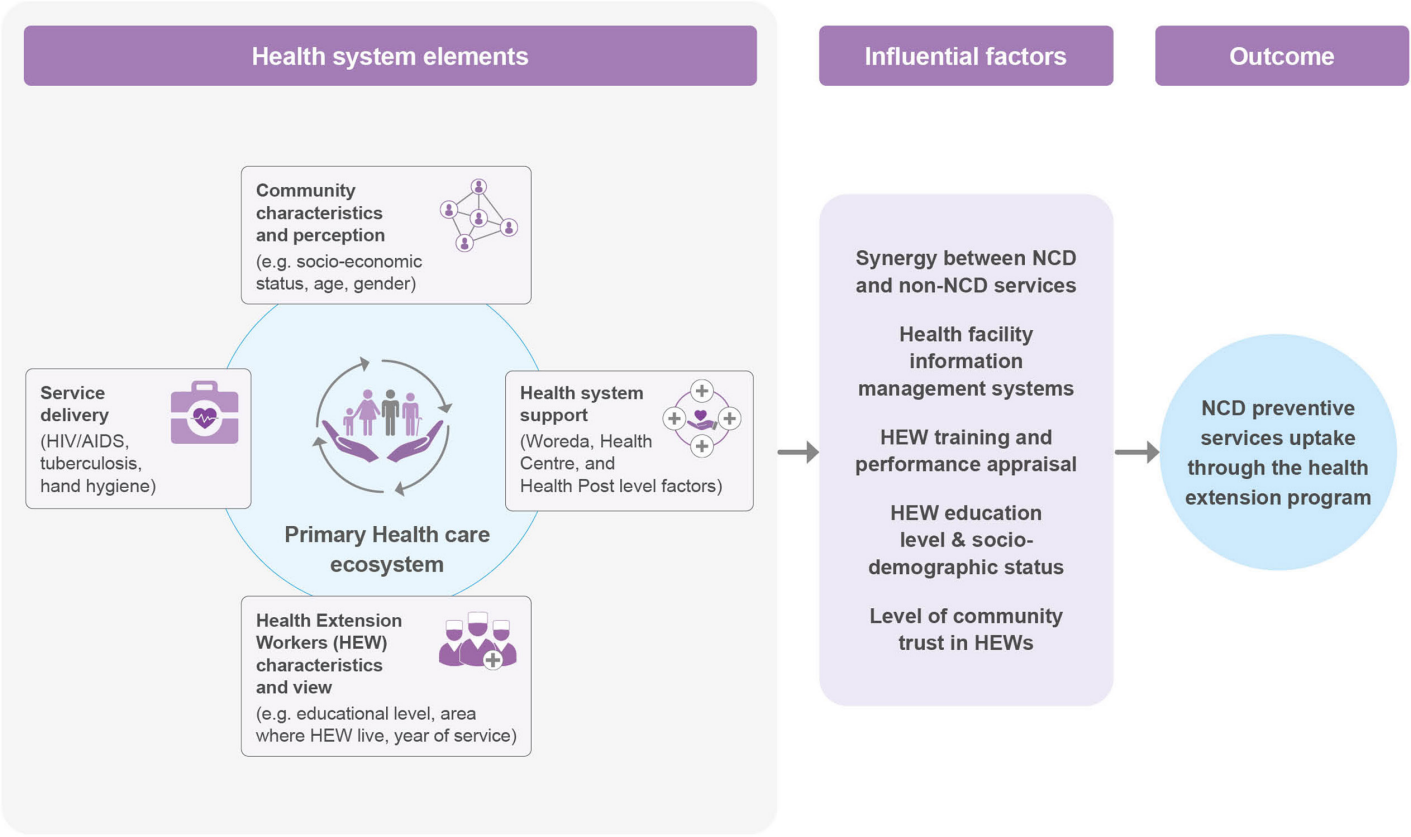
Factors affecting community health extension worker NCD prevention service in Ethiopia, adapted from Agrawal *et al*.^24^ HEW, health extension workers; NCD, non-communicable disease.

Accordingly, as shown in figure 1, our core covariates cover four main components: health system inputs and processes; HEW characteristics and perceptions towards the HEP; community resident attributes—such as age, gender, socioeconomic status (SES) and their perceptions towards HEWs and the HEP—and cross-programme effects from non-NCD services delivered through the HEP focussing on TB, HIV and environmental health services.

### Statistical analysis

The EHAS covered six different NCD interventions—namely, hypertension, heart disease, diabetes, kidney diseases, cervical and breast cancer—provided by HEWs through the HEP. Survey respondents were asked if they used any of the six NCD preventive services at least once in the year before the survey. The primary outcome variable was created by converting those responses into binary outcomes. We coded ‘1’ if residents reported using one or more preventive services at least once during the past 12 months and ‘0’ otherwise.

We applied a mixed-effects non-linear model with a logit link function and a random intercept at the woreda level to estimate the probability of NCD preventive service utilisation in the country.^25 26^ The random-intercept non-linear mixed-effects model suits the data’s hierarchical structure and the binary nature of the outcome variable. The random component addresses data clustering biases; and the effects of unobserved heterogeneity or omitted covariates measured at the woreda level. We fit the random variance at the woreda level because woredas play a pivotal role in Ethiopia’s multilayered health system. They are responsible for the planning, financing and monitoring health service delivery in their jurisdiction. They manage and coordinate the PHC units under their administrative control and have a technical link with the regional health bureaus and the zonal health department, requiring a high level of organisational and managerial competence.^27–29^

In this study, the models were estimated sequentially, starting with a null model (intercept-only model) that tested the null hypothesis that there was no between-cluster variation in NCD service delivery uptake. We then fitted a separate model, controlling only health system inputs and process variables. In the third stage, we added HEW-level attributes and perceived views on the current support system. Finally, we introduced variables capturing cross-programme effects, community characteristics and perception in the fourth and final model.

Resident perceptions were measured using 27 Likert-based questions. Out of these, we generated composite summary indices using principal component analysis (PCA). We retained factors with eigenvalues of unity or above, leading to two indices that broadly corresponded with the community’s views on HEP and towards HEWs competence to provide available services. Similarly, we aggregated the 10 Likert scale questions on HEWs’ perception of HEP and health system supports posed to HEWs’ into a single index using PCA. The analyses were conducted in Stata V.17 and weighted to account for non-response and design effects.^30 31^

### Patient and public involvement

No patients or public members were directly involved in the present study’s design as we used secondary data for the research. There are no plans to involve patients or the public in disseminating results.

## Results

### Community characteristics

The mean (SD) age of the sample population was 40.4 (13.81) years, and women were slightly over-represented (57.4%) in the sample. Twenty-two per cent of the resident population reported receiving prevention services on one or more of the six types of NCDs at least once during the past 12 months. Likewise, 18%, 12%, 16%, 13%, 12% and 13% reported having received hypertension, heart disease, diabetes mellitus, kidney disease and cervical and breast cancer prevention services from HEWs. In contrast, the uptake of other prevention services such as for HIV/AIDS and TB was much higher—89% and 84%, respectively. Receipt of hygiene advice was 47%—lower than these diseases but two-fold greater than for NCDs. However, mental health prevention service delivery was much lower than NCD services, 5.17% (table 1).

**Table 1:** Descriptive statistics of outcome variables, resident characteristics and perception for NCD prevention service, Ethiopian health extension programme, 2022

Sample community characteristics and perception (n=9680)	% or mean (weighted)
Community NCD prevention coverage* (%)	21.92
Respondents who have heard from HEWs about (%)	
Cervical cancer	12.89
Diabetes mellitus	16.48
Hypertension	18.02
Heart disease	12.33
Breast cancer	13.57
Kidney diseases	13.54
Respondents who heard from HEWs about tuberculosis	84.42
Respondents who heard from HEWs about HIV and AIDS	89.14
Respondents who heard from HEWs about hand hygiene	47.35
Respondents who heard from HEWs about mental illnesses	5.17
Gender (%), female	57.84
Wealth quintile index (%)	
Lowest	14.94
Lower	17.85
Middle	20.56
Higher	24.44
Highest	22.22
Composite index for community perception	
Community trust, acceptance and respect for HEWs (mean score)	0.89 (3.37)
Community perception towards HEWs competence to provide service (mean score)	0.62 (2.09)

*Percentage of respondents who have heard from HEWs about any type of NCD prevention service, out of the six, at least once in the past 12 months.

HEW, health extension worker; NCD, non-communicable disease.

### HEWs' characteristics

The mean (SD) age of HEWs was 26.3 (4.8) years, and an average HEW had served about 7.1 (4.7) years. Half (50 %) of the HEWs had level 4 qualifications, and one in five (22 %) reported having received short-term NCD training in the past year. Almost all HEWs (92 %) self-reported having the skill and being competent in measuring blood pressure (BP).

The majority (76 %) of HEWs were born or grew up in the same area as they worked in: 28.7% grew up in the same kebele, and 47.3% were from the same woreda, while less than a quarter—24.0 %—originated from outside. The majority (63 %) also currently reside in the same community that they serve (table 2).

**Table 2:** Descriptive statistics of health extension workers' characteristics and perception towards HEP, Ethiopian health extension programme, 2022

Health extension workers characteristics and perception	% or mean (weighted)
HEWs age (mean, years)	26.3 (4.8)
18–24 years	21.52
25–34	76.42
35–50	2.06
HEWs length of service year (mean, years)	7.1 (4.7)
0 –<5	23.29
5 –<10	19.69
10–16	57.02
Place where the HEW grew up (%)	
Outside of woreda	23.94
In the woreda	47.35
In the kebele	28.71
HEWs level of education (%)	
Level 1–3	49.89
Level 4	50.11
HEW’s marital status (%)	
Currently in union	74.64
Currently not in union	25.36
Area the HEWs' live (%)	
In nearby town	37.11
In the kebele	62.89
HEW who participated in NCDs short-term training (%)	21.86
HEW self-reported level of competence to measure BP (%)	91.94
HEWs’ perception towards HEP and health system support, composite index (mean score, SD)	−0.39 (1.02)

BP, blood pressure; HEW, health extension worker; NCD, non-communicable disease.

### Health system characteristics

Despite 99.9% of health centres saying they provided supervision visits to health posts, less than half (46.5%) of HEWs stationed at health posts reported receiving supportive supervision in the last 6 months (table 3). About three-fourth (73 %) of the health posts reported regularly compiling community profiles, while less than one-third of health centres (31 %) collected and interpreted NCD data from health posts. About two-fifth (36.5%) of health centres reported providing training for HEP coordinators in the last year. The same proportion, 37%, of health centres also reported providing training for HEWs in the past 2 years (table 3).

**Table 3:** Descriptive statistics of health system inputs for NCD prevention service, Ethiopian health extension programme, 2022

Sample characteristics (n=9680)	% or mean (weighted)
**Health system inputs and process**	
Health post compile community (kebele) information profile (%)	73.38
Health centre’s HEP coordinator received training on HEP packages (%)	36.49
HEWs’ involvement in community activities other than HEP (%)	82.16
Health post had received supportive supervision from woreda health office in the last 6 months (%)	46.49
Health centres provide supervision to health post (%)	99.89
Health post with blood pressure (BP) measurement service (%)	62.20
Most recent performance assessment made for HEW by (Month/s ago) (mean, SD)	1.96 (2.15)
Community members involved in the performance assessment of HEWs	
No	84.24
Yes	15.76
Health centres provide training for HEWs in the last 2 year (%)	37.37
Health centres collect NCD reports from health posts (%)	31.09

HEW, health extension worker; NCD, non-communicable disease.

### Determinants of utilisation of NCD prevention services

Online supplemental file 1 shows the results of the multilevel analysis (models 0–3). The null model, represented by model 0, has no covariates; it provides between-cluster variance (ie, between woreda). Model 1 captures the effects of health systems inputs on predicting NCD preventative uptake without controlling for additional variables. Model 2 simultaneously estimates the associations between health system-level and HEW-level attributes and NCD service uptake. The final model, model 3, portrays the effects of health system-level, health worker-level and person-level characteristics on NCD utilisation.

10.1136/bmjgh-2022-009025.supp1 Supplementary data

**Figure 2:**
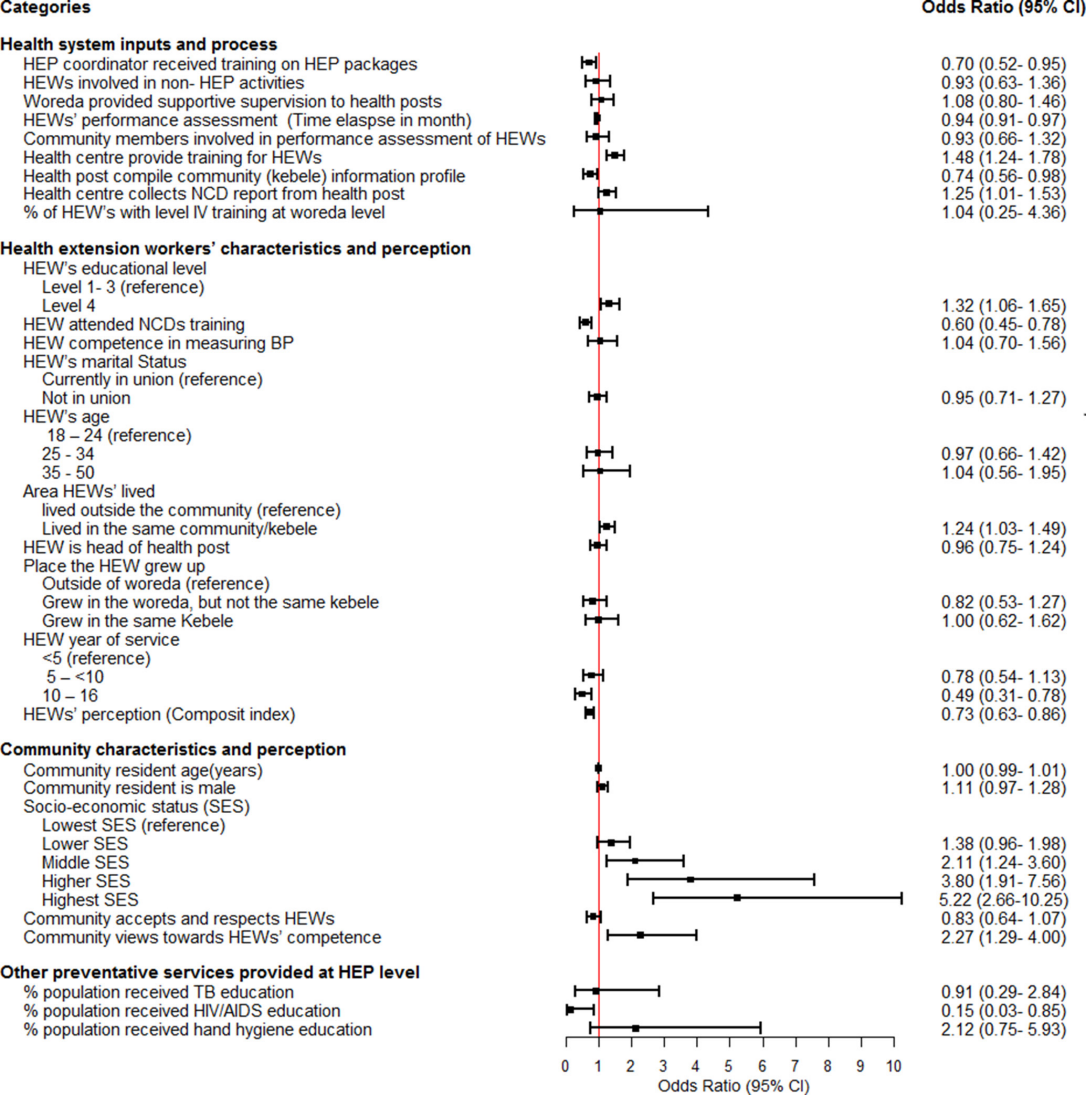
Forest plot: health system, health worker and community characteristics effects on receiving NCD prevention service, Ethiopia: 2022. HEW, health extension worker; NCD, non-communicable disease; SES, socioeconomic status; TB, tuberculosis.

While all the three models with covariates (models 1–3) generally reveal similar patterns, adding all three sets of covariates together into the same model in model 3 resulted in additional significant variables than those identified in models 1 and 2. Our final model, model 3, is presented in a forest plot in figure 2.

The forest plot shows that the odds of receiving NCD prevention service by a community member increased by up to 25% (OR=1.25, CI 1.01 to 1.53) if health centres routinely gathered NCD report from health posts and by up to 48% (OR=1.48, CI 1.24 to 1.78) if they provided training to HEWs. These results are similar to those observed in models 1 and 2. Our findings also showed that if the HEWs have a level IV qualification and live in the same community they serve, the odds of NCD utilisation in the community increase by about 32% (OR=1.32, CI 1.06 to 1.65) and 25% (OR=1.24, CI 1.03 to 1.49), respectively. However, long years of service (OR=0.49 CI 0.31 to 0.78) and perceived lack of health system support by HEWs (OR=0.73 CI (0.63 to 0.86) adversely impact coverage of NCD prevention uptake. Similarly, delays in conducting routine HEW performance assessments had an adverse effect, as were the health centre’s involvement in providing HEP packages training for HEP coordinators. Each month’s delay in conducting HEWs performance reviews lowered community uptake of NCD prevention services by 6% (OR=0.94, CI 0.91 to 0.97).

There was a negative association between programme performance on HIV/AIDS and community uptake of NCD preventive services (OR=0.15, CI 0.03 to 0.85). We found a strong socioeconomic gradient with NCD preventive service utilisation. Those in the highest, higher and middle SES categories had a fivefold (OR=5.22, CI 2.66 to 10.25)), 3.8-fold (OR=3.80, CI 1.91 to 7.56) and twofold (OR=2.11, CI 1.24 to 3.60)) higher uptake of NCD services than those in the lowest SES category. However, other sociodemographic attributes such as age and gender of residents did not affect the utilisation of NCD preventive services.

The variance component was statistically significant across the four models. The addition of health system, HEW and community-level characteristics have increased rather than lessened the residual between-cluster variance. With a between-cluster variance of 1.85 (1.07 to 3.20), p<0.001) shown for the final model, the estimated intraclass correlation was 0.36, meaning that a third of the variance in NCD service uptakes was due to the variations between woredas.

## Discussion

For close to two decades, Ethiopia has implemented a PHC-focused HEP to increase coverage of essential services and promote health outcomes.^32^ While studies have examined the health impacts of the HEP, its effect on NCD preventive service uptake and the competition and complementarity with other existing programmes, if any, remain unexplored.^4 33 34^

Our results showed that utilisation of NCD prevention services through the HEP remains limited. Less than a quarter (22%) of the study population had accessed NCD services 12 months before the survey, and service uptake was significantly associated with the SES of residents. Those in the highest, higher and middle SES categories had greater access to NCD preventive services than residents in the lower and lowest SES categories. A previous study in Ethiopia also showed a similar finding for the country’s other existing services—mothers from higher income families were more likely to visit health posts than lower income families.^35^ This is concerning, given that the HEP itself was explicitly designed to improve access for underserved groups and address existing health inequalities.^7^

Our study identified several factors operating at the health system level that demonstrate the complementarity and competition between NCD and other essential preventive services.

### Effects of district-level health system inputs and processes

We found that systematic collection of NCD reports from health posts facilitates the uptake of NCD services. These findings are consistent with previous studies where data reporting and data utilisation played a crucial role in improving health service delivery at the community level.^24^ However, we found that health posts’ engagement in compiling community profiles adversely affects coverage of NCD services, which could indicate potential competition between time spent on core activities versus other commitments. A time–motion study conducted in Ethiopia showed that recordkeeping, reporting and managing family folders consume more than a tenth of available time,^36^ which was considered substantial and believed to have had adverse effects on new HEP packages.

Health system inputs, such as health information management systems and programmatic processes like HEW training, supportive supervision, and performance appraisal, are essential for developing problem-solving skills and fostering quality of care practices within service delivery settings.^17 24^ Our result showed a strong link between HEW performance evaluation practices and NCD prevention services, with delays in conducting assessments having a significant adverse effect on NCD uptake. While a qualitative study in Ethiopia suggests that PHC services receiving greater attention are more likely to be implemented,^23^ our findings demonstrate complementary effects, as none of the health system factors—such as overall HEW training, supportive supervision, and performance appraisal—captured in our study was explicitly aimed at NCDs.

However, the finding on training, which showed attendance by HEP coordinators as having a negative impact on NCD uptake, is contrary to conventional beliefs. In general, attending training programmes is expected to improve know-how, enhance health workers’ performance and a programme coordinator’s capacity to deliver services.^17 24^ While our data do not explicitly capture the type, quality or duration of training received by programme coordinators, the contents of most training opportunities in Ethiopia are known to focus on HIV, MCH and malaria due to the high priorities accorded to those services.^7^ These may explain the unexpected relationship between HEP coordinator training and NCD service utilisation observed in the current study.

In the current study, the involvement of woreda administrators in HEWs supervision also did not show any significant association with improving NCD prevention service coverage. While some studies found results like ours,^37^ the absence of association in the current research is contrary to evidence that supportive supervision is critical for programme success and effective NCD service delivery by CHWs.^14 17 24^ As evidence suggests, supervision needs to be linked to performance goals, targeted to a specific group and focused on particular knowledge and skillset to have the desired effect.^17^ It also requires a well-trained staff who have subject matter know-how and are able to guide and support HEWs. The absence of a strong relationship in the current study may be attributed to the lack of quality supervision and insufficient attention to NCD services during supervisory visits. Previous research on Ethiopia’s NCD programme at the PHC level indicated that supervisors had minimal knowledge of NCDs while the supervision system itself lacked NCD focus.^23^ Such a lack of a deeper understanding of NCD knowledge among supervisors is likely to lead to a greater emphasis on supporting programmes, they were familiar with and trained on; hence the insignificant association with NCD observed in the present study.

### Effect of HEW characteristics and perception towards lack of health systems support

The role of HEW attributes in improving access and coverage of essential health services has been documented in Ethiopia, especially for MCH, HIV, TB and malaria programmes.^4 33 34 38 39^ Even though evidence on CHWs and NCDs is still evolving, the few studies elsewhere highlight the potential role of CHW in delivering NCD interventions.^13–18^ In line with these studies, we found that NCD uptake was positively and significantly associated with HEW qualification and living arrangements in the communities they served. Uptake of NCD preventive services tended to be higher if the HEW had level IV qualifications and lived in the same community.^14^ This may be because, in Ethiopia, HEWs with level IV qualifications, as opposed to level III or below, receive NCD-focused content as part of their additional year-long training on the HEP.

However, unlike the case for family planning, reproductive health and HIV/AIDS services, community uptake of NCD preventive services was not affected by other demographic attributes of HEWs, such as age, gender or marital status. On the other hand, their service year and perceived lack of support from the health system had an adverse impact on NCD programme uptake. This demonstrates yet another complementary effect—in that HEWs’ perception was not specific to NCDs but cut across other services. Although work experience generally improves performance, the negative association observed for service years in the present study may be attributed to frustration with the system. The absence of career advancement opportunities also limits long-term commitment among health workers and encourages frequent job changes.^14^ In Bangladesh and China, where CHWs received the required health system support, such as training and necessary equipment, they were confident in delivering essential NCD-related services. In contrast, in Viet Nam and Nepal, where CHWs were not adequately trained and did not have the necessary equipment, they were less confident and lacked the skills to deliver NCD-related services.^14^

In addition, we observed that HEWs’ participation in short-term NCD training did not improve community NCD service uptake. The negative association between NCD service coverage and short-term training remained even after we controlled for potential interaction with HEWs’ level of qualification. The negative effect was, in fact, more substantial for those with level III qualifications or below, indicating that skill upgrading through short-term training alone may not solve the problem.^7^ This may be so because short-term training for CHWs often focus on a limited set of skills (ie, BP measurement for hypertension) rather than comprehensive training that covers several common NCDs (ie, hypertension, diabetes and cancer).^14^ The finding may also further suggest another complementary effect—the overall low level of competence of HEWs may itself have limited their ability to benefit from short-term NCD training while at the same time adversely affecting the quality-of-service provision at the community level.^7^

### Effect of community characteristics and perception towards HEP and HEWs

Several studies have shown that community-level NCD service provision depends on the broader SES of the community, their perception of health programmes in the community and CHWs’ capacity to deliver mandated services.^14 20^ Our findings suggest that such complementarity as the uptake of NCD preventive services was higher if residents already hold favourable views towards the HEP, trust HEWs and their competency in providing services. This finding is similar to previous studies in Ethiopia for other existing HEP services, which demonstrated a positive association between programme success and community perception towards HEWs’ role and the HEP more generally.^19 20^ These studies showed that maternal health outcomes tend to be positive if HEWs have a good relationship with the local community and are trusted with their skill set.^19 20^ Community trust and acceptance of health workers and positive views on programmes under implementation create a conducive work environment for CHWs and contribute to their retention, motivation, performance, accountability and receiving support from the community.

### Effect of other programmes on NCD service delivery

There is growing evidence that integrating NCD care with other existing services is feasible and an opportunity to achieve Universal Health Coverage.^40 41^ Should integration prove to be effective, it can increase coverage of NCD services and potentially improve health outcomes for those with NCDs. However, our study showed no association (neither complementary nor competitive) between TB and hand hygiene programmes and NCD coverage. In contrast, HIV/AIDS programme performance had statistically significant adverse (ie, competitive) effects. The negative association between HIV/AIDs programme performance and NCD coverage supports the view that integration with new programmes could, competitively, spread resources too thinly in the face of a dwindling funding opportunity for HIV programmes.^42^ This, in turn, may jeopardise the success of HIV services and may result in worse health outcomes for people living with HIV and little benefit for those living with NCDs.

### Strengths and limitations of the study

Finally, a few caveats around the research are in order. The data used in our study were collected in 2019, but much has changed since in Ethiopia. The ongoing civil war in the country is likely to have shifted the attention and focus of the government, reoriented policy and funding priorities and disrupted health services previously available to the population. Moreover, like the rest of the world, Ethiopia is in the middle of the COVID-19 pandemic, which also impacted priorities and service coverage across the health sector. Findings from the present study and application of results for designing future programmes, thus, need to take these events and developments into account.

Another limitation of our analysis is the cross-sectional nature of the data, as it only shows association and does not necessarily control for lag effects. For example, the opposite associations between NCD specific short-term training for HEW and NCD prevention service uptake could be mere reflections of lack of adequate observational time and teething effects related to recruitment and targeting. Second, the competing relationship between NCD preventive services and HIV/AIDS is likely to evolve. A complete picture of such dynamic relationships requires much more than a cross-sectional evaluation design can provide. In addition, the inclusion of other existing services, such as MCH, in the analysis could have provided additional insight into the synergy between NCD and other HEP services. Our data had only a limited number of comparable information on existing HEP preventive services. Hence, future evaluations should adopt longitudinal designs to address these shortcomings.

Despite the limitations, the study also has several strengths. It is the most extensive programme evaluation of HEP in Ethiopia and probably one of the largest CHWs evaluations in Africa. It is also the first-ever study globally to empirically explore the complementarity and competition between NCD and other essential preventive services.

## Conclusion

Results on a range of complementary effects point to the need for an overall strong HEP capable of delivering existing services as a prerequisite for successfully integrating new (in this case, NCD) services.^43^ For example, strengthening overall training and supervision and building trust between the community and the HEWs are essential for a successful HEP, just as they are for individual services within the HEP and integrating new ones such as NCD preventive services. However, integration efforts also require a carefully balanced approach, so that the success already recorded for some existing programmes is not lost.

10.1136/bmjgh-2022-009025.supp2 Supplementary data

## Data Availability

Data may be obtained from a third party and are not publicly available. All relevant data contributing to the findings are within the paper and in Supplementary table 1. As secondary data users, we are restricted by data sharing policy and ethical clearance to share additional data. All request for the original data should be directed to the data custodian, the MERQ Consultancy.
