# Supplementary material for: Exploring complementary and competitive relations between non-communicable disease services and other health extension programme services in Ethiopia: a multilevel analysis
Source: BMJ Glob Health. 2022 Jun 23;7(6):e009025. doi: 10.1136/bmjgh-2022-009025 (PMC9226884; doi:10.1136/bmjgh-2022-009025)
Supplement: Supplementary data [file bmjgh-2022-009025supp001.pdf]

Supplementary table 1: Health system, health worker and community characteristics effects on receiving NCD prevention service, Ethiopia: 2022.

Fixed and random effects	Null model OR [95% CI]	Model 1 OR [95% CI]	Model 2 OR [95% CI]
**Health system inputs and process**			
HEP coordinator received training (Ref=No)			
Yes		1.04 [0.80, 1.36]	0.89 [0.66, 1.22]
HEWs' involvement in non- HEP activities (Ref = No)			
Yes		0.99[0.87, 1.15]	0.93 [0.69, 1.26]
Involvement of woreda in HEWs supervision (Ref = No)			
Yes		1.14[0 .89, 1.46]	1.14 [0.84, 1.55]
Time elapse since the last HEWs' performance assessment (PA) was undertaken by the health centre (Month/s)		0.94[0.90, 0 .99] *	0.95 [0.91, 0 .99] *
Community members involvement in PA for HEWs (Ref = No)			
Yes		0.84[0.64, 1.11]	1.07[0.78, 1.46]
HC provides training for HEWs (Ref = No)			
Yes		1.34 [1.08, 1.65] **	1.27[0.97, 1.67]
HP compiles kebele profile (Ref = No)			
Yes		0.90[0.69, 1.17]	0.71 [0.54, 0.93] *
HC collects NCD report from HP (Ref = No)			
Yes		1.17[0.91, 1.48]	1.51[1.16, 1.98] **
% of HEW's with level IV training in the woreda		1.93[0.68, 5.47]	1.36[0.42, 4.35]
**Health extension workers' characteristics and perception**			
HEW's educational level (Ref = Level 1- 3)			
Level 4			1.51[1.19, 1.91] ***
HEW has attended NCDs shOort-term training (Ref = No)			
Yes			0.67[0.48, 0 .92] *
HEW feels competence in measuring BP (Ref = No)			
Yes			0.95[0.60, 1.49]
HEWs' perception towards health system supports available to them (composite index)			0.77[0.66, 0.91] **
HEW's marital Status (Ref = currently in a union)			
Currently not in union			0.82 [0.68, 0.99]
HEW's age (Ref = 18 – 24)			
25 – 34			0.89 [0.58,1.37] *
35 – 50			1.17[0.57, 2.40]
Area HEWs' lived (Ref = lived outside the community)			
Lived in the same community/kebele			1.21[0.99, 1.47]
HEW is head of the health post (Ref = No)			
Yes			0.93[0.70, 1.21]
Place the HEW grew up (Ref = outside of woreda)			
Grew in the woreda, but not the same kebele			0.97[0.69, 1.36]

Grew in the same Kebele			0.97[0.63, 1.49]
Duration of service for the HEW (in years) (Ref = Under five years)			
5 – <10			0.79[0.55, 1.15]
10 – 16			0.56[0.35, 0.91] *
**Community characteristics and perception, and other preventative services provided at HEP level**			
Age of participant (in years)			
Gender (Ref = Female)			
Male			
Socio-economic status(SES) (Ref = Lowest SES)			
lower SES			
Middle SES			
Higher SES			
Highest SES			
Community accepts and respects HEWs (composite index)			
Community has favourable views towards HEWs' competence			
% population received health education on TB from HEW			
% population received health education on HIV/AIDS from HEW			
% population received health education on hand hygiene from HEW			
Constant		0.08[0 .15, 0.44] ***	0.22 [0.04, 1.29] **
**Random effect**			
District/Woreda level Variance	1.25 [0.66, 0.37]	1.26[0.69, 2.29]**	1.44[0.89, 2.36] ***
* p<0.05, ** p<0.01, *** p<0.001			
