# Supplementary material for: Exploring complementary and competitive relations between non-communicable disease services and other health extension programme services in Ethiopia: a multilevel analysis
Source: BMJ Glob Health. 2022 Jun 23;7(6):e009025. doi: 10.1136/bmjgh-2022-009025 (PMC9226884; doi:10.1136/bmjgh-2022-009025)
Supplement: Supplementary data [file bmjgh-2022-009025supp002.pdf]

Appendix S1 – Author Reflexivity Statement

Title: Exploring complementary and competitive relations between non-communicable disease (NCD) services and other health extension program services in Ethiopia: a multilevel analysis

1. How does this study address local research and policy priorities?

The paper on the Ethiopian health extension program (HEP) was designed to investigate the utilization of NCD preventive services through the HEP and explore the complementary and competitive relations between NCD services and other health extension program services in Ethiopia. This study's recommendations will assist policymakers in effectively integrating NCD services into the country's primary health care system.

2. How were local researchers involved in study design?

The study was part of a PhD project for the principal investigator, AGT. AGT has been working as a researcher at Mekelle University, Ethiopia, and has moved to a high-income country for further studies. AT and FWF are local researchers with extensive in-country experiences. YK is originally from the study country and has experience as a local researcher in Ethiopia. The study also involves high-income country researchers with expertise in conducting and supervising research collaborations involving Ethiopia and other low and middle-income countries (DP, RJ and SA).

3. How has funding been used to support the local research team?

There was no specific fund dedicated to this study. However, AGT is supported by a UNSW Scientia Scholarship and a top-up scholarship from the George Institute for Global Health. Further funding information for all authors can be found in the main paper.

4. How are research staff who conducted data collection acknowledged?

As secondary data users, we acknowledged the organization that provided us with the data; please see the acknowledgement section of the paper.

5. Do all members of the research partnership have access to study data?

All members of the partnership have access to data.

6. How was data used to develop analytical skills within the partnership?

YK guided AGT on the data analysis process. All authors were involved in the analytical interpretation of the data.

7. How have research partners collaborated in interpreting study data?

Monthly meetings were held with the research team to discuss the data analysis process, the preliminary findings, and the data interpretation.

8. How were research partners supported to develop writing skills?

As an early career researcher and a PhD candidate, AGT was supported by senior academics and researchers involved in this study to develop and refine their writing skills.

9. How will research products be shared to address local needs?

The paper will be published in an open-access journal, making it accessible for local researchers interested in similar areas in Ethiopia and beyond. The research team also has a post-publication dissemination plan, including the dissemination of key findings and recommendations to relevant stakeholders in Ethiopia.

10. How is the leadership, contribution and ownership of this work by LMIC researchers recognized within the authorship?

Author AGT led the study, and this has been recognized as being a first author. We have strong representation from researchers who are based in LMICs (AT and FWF) and are supported by others based in high-income countries.

11. How have early career researchers across the partnership been included within the authorship team?

We have included an early career researcher (AGT) within the authorship team.

12. How has gender balance been addressed within the authorship?

Tesema AG, *et al. BMJ Global Health* 2022; 7:e009025. doi: 10.1136/bmjgh-2022-009025

Two of the authors (AGT and RJ) are female.

13. How has the project contributed to training of LMIC researchers?

The authorship team is primarily composed of senior researchers. The authors from LMIC are senior researchers. And AGT is currently a research scholar working toward her terminal degree.

14. How has the project contributed to improvements in local infrastructure?

This project has not directly contributed to improvements in local infrastructure; however the findings have several recommendation for strengthening Ethiopia's Health Extension Program including increased investment in health facility capacity to manage non-communicable diseases.

15. What safeguarding procedures were used to protect local study participants and researchers?

We used secondary data for this study. Hence, this question is not directly applicable in the current context.

Tesema AG, *et al. BMJ Global Health* 2022; 7:e009025. doi: 10.1136/bmjgh-2022-009025
